# Seroprevalence of SARS-CoV-2 IgG antibodies and risk factors in health care workers at an academic medical center in Boston, Massachusetts

**DOI:** 10.1038/s41598-021-89107-5

**Published:** 2021-05-06

**Authors:** Yachana Kataria, Manisha Cole, Elizabeth Duffy, Kyle de la Cena, Elissa M. Schechter-Perkins, Tara C. Bouton, Martha M. Werler, Cassandra Pierre, Elizabeth J. Ragan, Sarah E. Weber, Karen R. Jacobson, Chris Andry

**Affiliations:** 1 Clinical Chemistry, Department of Pathology and Lab Medicine, Boston Medical Center, 670 Albany St., Boston, MA 02118 USA; 2 Department of Pathology and Laboratory Medicine, Boston University School of Medicine, Boston, MA USA; 3 Department of Emergency Medicine, Boston University School of Medicine/Boston Medical Center, Boston, MA USA; 4 Section of Infectious Diseases, Boston Medical Center, Boston, MA USA; 5 Department of Epidemiology, Boston University School of Public Health, Boston, MA USA

**Keywords:** SARS-CoV-2, Epidemiology

## Abstract

Healthcare workers (HCWs) are at an increased risk of severe acute respiratory syndrome coronavirus 2 (SARS-CoV-2), a novel virus that causes Coronavirus Disease (COVID-19). We aim to assess the seroprevalence of SARS-CoV-2 IgG among healthcare workers and compare risk-factors between seropositive and seronegative HCWs. In this observational study, serum samples were collected from HCWs between July 13th to 26th, 2020 at Boston Medical Center (BMC). Samples were subsequently tested for SARS-CoV-2 IgG antibody using the Abbott SARS-CoV-2 IgG assay. Participants also answered a questionnaire capturing data on demographics, history of COVID-19 symptoms, occupation, infection prevention and control measures. Overall, 95 of 1743 (5.5%) participants tested positive for SARS-CoV-2 IgG. Of these, 1.8% of the participants had mild or no COVID-19 symptoms and did not require a diagnostic test. Seropositivity was not associated with gender, occupation, hand hygiene and personal protective equipment (PPE) practices amongst HCWs. However, lack of physical distancing among health care workers in work areas and break room was associated with seropositivity (p = 0.05, p = 0.003, respectively). The majority of the HCWs are negative for SARS-CoV-2 IgG. This data highlights the need to promote infection prevention measures, and the importance of distance amongst co-workers to help mitigate infection rates.

## Introduction

Healthcare workers (HCWs) are at an increased risk for severe acute respiratory syndrome coronavirus-2 (SARS-CoV-2), a novel virus that causes Coronavirus Disease 2019 (COVID-19). COVID-19 has infected nearly 7.75 million and caused the deaths of over 214,000 people in the United States as of October 11th, 2020. As of July 16th, 2020, 100,570 HCWs with confirmed COVID-19 and 641 deaths were reported to the United States Centers of Disease Control (CDC)^1,2^. This comprised 22% of cases reported to the CDC^3^. It is important to understand the prevalence and risk factors amongst HCWs as it can inform infection prevention and control measures.

The first case of COVID-19 in Massachusetts was reported on February 1st, 2020^4^. The number of cases in the state grew quickly due to a widely attended scientific conference, considered to be a superspreading event, and Massachusetts experienced a surge in mid-April, 2020^5^. Boston Medical Center (BMC), a 514-bed academic medical center, historically the safety-net hospital for the city of Boston, experienced a steep rise in cases during early April to mid-May. At peak, BMC, averaged over 30 COVID-19 admissions per day with a hospital census of over 230 SARS-CoV-2 positive patients. At peak our institution experienced a 53% positivity rate for diagnostic testing; by July, positive test rates were less than two percent.

Seroprevalence studies can assist in estimating the proportion of a population that has been infected. It provides a better estimate of population level-data by capturing individuals with mild or no symptoms and others who never underwent diagnostic testing. This is especially important for COVID-19 because people with asymptomatic infections are thought to make up a majority of SARS-CoV-2 infections, but are less likely to present for diagnostic testing^6^. Assessing the cumulative prevalence is critical to understanding disease transmission rates.

HCWs spend a significant amount of time in a high-risk setting. Once infected, they can spread SARS-CoV-2 to patients, colleagues, and members of the larger community. Literature suggests the general population has a wide seroprevalence range, between 2.7–16.6%, and HCW range from 1.3–22.0%^2,7–13^. A recent report from Asian countries reported that HCWs constituted over 20% presumptive occupation related cases^14^. In July, the Boston community prevalence was reported to be over 116,000 confirmed and probable cases^15^. However, the disease burden amongst HCWs in Boston remains unknown. It is important to understand disease prevalence and characteristics amongst HCWs as it can identify areas or personnel that are at increased risk. It can also inform infection control policy in the hospital setting to mitigate infection rates.

We aim to assess the seroprevalence of SARS-CoV-2 among healthcare workers at BMC and compare characteristics, including demographics, occupation, COVID-19 symptoms, and infection prevention and control measures taken between seropositive and seronegative HCWs.

## Methods

### Study design and study population

We conducted a cross-sectional study at BMC (July 13th to July 26th, 2020) to detect SARS-CoV-2 Immunoglobulin G (IgG) antibodies in HCWs. Eligible participants worked at BMC during the initial COVID-19 surge at BMC (March 13th to May 31st, 2020). BMC, located in Boston, Massachusetts, has approximately 7442 employees. This project was approved by the Institutional Review Board at BMC. All methods were performed in accordance with relevant guidelines and regulations.

All eligible study participants were offered SARS-CoV-2 IgG antibody test. Eligible HCWs were at least 18 years old and worked physically on the BMC campus during the study period. Participants were recruited via a multi-pronged approach including email communications, physical flyers in employee-only spaces on campus, advertisement on the hospital’s internal website, announcements at a hospital-wide town hall, and at department-level meetings.

Potential participants opted-in to the study by following the link in recruitment materials to a REDCap (Research Electronic Data Capture) survey^16^. Using this platform, participants filled out a pre-screening questionnaire, and completed an electronic informed consent form if deemed eligible. Participants then provided the following information in online self-administered surveys: sociodemographic data (age, sex, height, weight), occupation, self-report of COVID-19 related symptoms, known COVID exposure, prior SARS-CoV-2 RT-PCR test result, if any, and infection prevention and control measures. All SARS-CoV-2 IgG levels were obtained from participants between July 13th–26th, 2020. A subset of participants who were also eligible for an additional study were scheduled for a phone-based questionnaire with the same questions captured by the electronic version. Consent and questionnaire were available in English, Spanish and Haitian-Creole, reflecting the predominant languages spoken by BMC staff.

### RT-PCR measurement

BMC HCWs with suspicion for COVID-19 were tested for SARS-CoV-2 infection in accordance with hospital policy via a RT-PCR assay. Study participants self-reported their SARS-CoV-2 RT-PCR test history and corresponding test result.

### SARS-CoV-2 antibody measurement

IgG antibody analyses were performed by the clinical pathology laboratory at BMC. Serum samples were run on the Abbott Architect i2000 Instrument using the Abbott SARS-CoV-2 IgG assay per the manufacturer’s instructions (SARS-CoV-2 IgG; Abbott Laboratories, Abbott Park, IL). This assay is a chemiluminescent microparticle immunoassay for detection of IgG antibody in human serum against the SARS-CoV-2 nucleoprotein. Samples were interpreted as positive (index value > = 1.4) or negative (index value < 1.4) based on the index values reported by the instrument. Qualitative results were used in the analyses.

Low and high level of quality control (QC), as supplied by the manufacturer, were run twice daily during the study period. The inter-day coefficient of variation (CV) for low and high levels were 14.30%, and 2.78%, respectively.

### Statistical analysis

All questionnaire data were collected in REDCap. Non-English questionnaire data responses were manually translated to English. Categorical variables are presented as counts. We tested the association between serology status and variables of interest with either a Chi-Square or Fisher’s Exact test. Missing data ranged from 0.1 to 5.3%. If the missing data for any variable was less than five percent, it was excluded from analysis. Otherwise, it is indicated in the tables. Risk ratios (RR) and 95% confidence Intervals (CI) were calculated using the epitools add-on package in R. A P-value of < 0.05 was considered statistically significant, and all tests were two-sided. Analyses were performed in R Version R-1.3.1056 (R Foundation for Statistical Computing)^17^. Graphical representations were performed on GraphPad Prism software version 9.0 (GRAPH PAD software Inc, California, USA).

## Results

### Baseline characteristics

**Figure 1:**
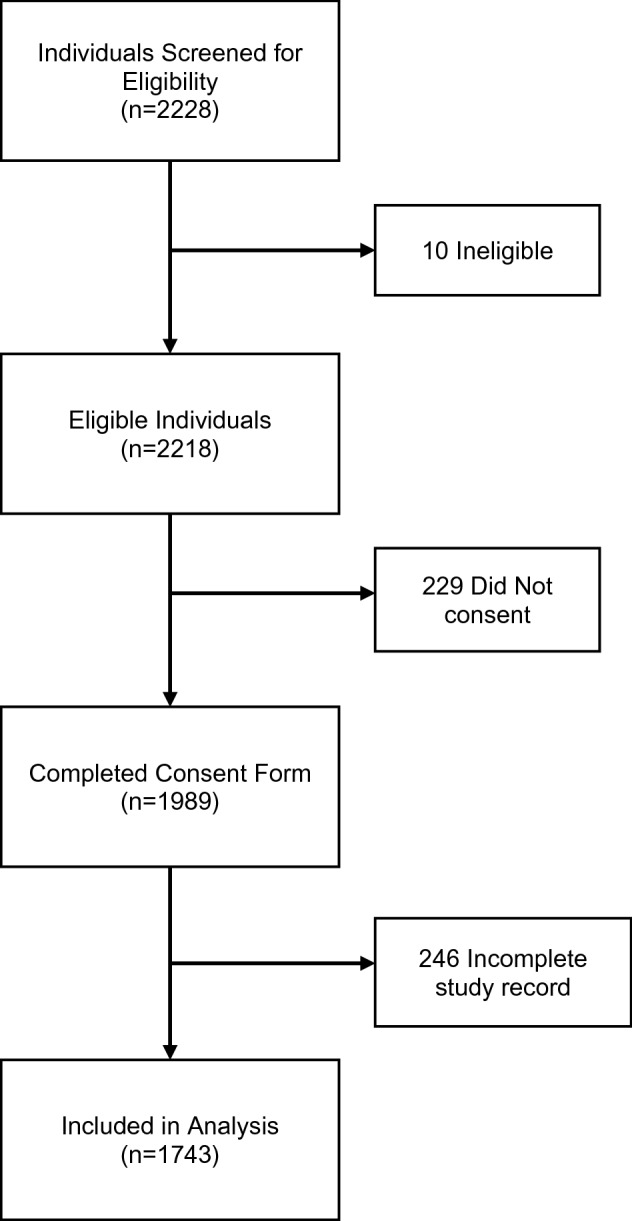
Study enrollment.

All BMC employees that worked on campus during the surge were invited to participate, with 2228 completing the initial screening agreement. Ten participants were ineligible as they did not work on campus during the first surge or were not healthcare providers at BMC. Of the 2218 eligible HCWs, 1989 (89.6%) individuals went on to provide informed consent. Of those who provided informed consent, 246 (12.3%) individuals did not complete the survey and/or get a blood draw. A total of 1743 individuals with complete data were included in the analysis (Fig. 1).

The mean age of participants was 38.9 years (SD 15.2 years). Participants were predominantly female (74.8%), half self-reported as overweight or obese (49.5%). A majority (74.5%) of the participants identified as White. The remaining identified as either Asian, Black, or Hispanic (9.2%, 8.1%, and 8.2%, respectively).

Most of the enrolled participants were nurses (n = 716, 41.1%) or physicians (n = 523, 30.0%). Administrative staff and patient/non-patient facing allied-health professionals comprised of 8.3%, 12.7% and 6.4%, respectively, of the total participants (Table 1). Facilities management and support services, which encompass HCWs from environmental services, housekeeping, valet, public safety represented 1.4% of the total participants.

**Table 1:** Study demographics.

Variable	Total n (column %)	SARS-CoV-2 IgG Status		p-value^†^ (all data)	RR (95% CI) (all data)
		Positive n (row %)	Negative n (row %)		
Total	1743	95 (5.5%)	1648 (94.5%)		
**Sex**				0.55^†^	
Female	1304 (74.8%)	75 (5.8%)	1229 (94.2%)		1 (referent)
Male	432 (24.8%)	20 (4.6%)	412 (95.4%)		0.79 (0.49–1.29)
Nonbinary/third gender	3 (0.2%)	0	3 (100%)		0 (0.00–NaN**)
**Age (years)**				p < 0.001^†^*	
< 20	93 (5.3%)	16 (17.2%)	77 (82.8%)		1 (referent)
20–29	334 (19.2%)	17 (5.1%)	317 (94.9%)		0.28 (0.15–0.54)*
30–39	534 (30.6%)	22 (4.1%)	512 (95.9%)		0.23 (0.12–0.42)*
40–49	322 (18.5%)	15 (4.7%)	307 (95.3%)		0.26 (0.13–0.50)*
50–59	294 (16.9%)	20 (6.8%)	274 (93.2%)		0.38 (0.20–0.70)*
60–69	155 (8.9%)	5 (3.2%)	150 (96.8%)		0.18 (0.07–0.47)*
> 69	11 (0.6%)	0	11 (100%)		0.00 (0.00–NaN**)
**BMI**				0.02^†^*	
Underweight	30 (1.7%)	0	30 (100%)		0.00 (0.02–5.56)
Normal	843 (48.4%)	39 (4.6%)	804 (95.4%)		1 (referent)
Overweight	529 (30.3%)	25 (4.7%)	504 (95.3%)		1.00 (0.62–1.71)
Obese	335 (19.2%)	30 (9.0%)	305 (91.0%)		1.97 (1.25–3.32)*
**Hispanic/LatinX**				0.05^†^	
Yes	143 (8.2%)	13 (9.1%)	130 (90.9%)		1.79 (1.02–3.14)*
No	1594 (91.5%)	80 (5.0%)	1514 (95.0%)		1 (referent)
**Race**				0.54^†^	
Asian	160 (9.2%)	10 (6.3%)	150 (93.8%)		1.27 (0.66–2.42)
Black	142 (8.1%)	11 (7.7%)	131 (92.3%)		1.57 (0.85–2.92)
White	1299 (74.5%)	63 (4.8%)	1236 (95.2%)		1 (referent)
Native American/Pacific Islander	7 (0.4%)	0	7 (100%)		0.00 (0.00–NaN**)
Other	112 (6.4%)	5 (4.5%)	107 (95.5%)		0.91 (0.37–2.20)
**Smoking**				0.33^†^	
Yes	47 (2.7%)	4 (8.5%)	43 (91.5%)		1.55 (0.59–4.04)
No	1675 (96.1%)	91 (5.4%)	1584 (94.6%)		1 (referent)
**Occupation**				0.0028^†^*	
Administrative	145 (8.3%)	7 (4.8%)	138 (95.2%)		1 (referent)
Allied health—non-patient facing	112 (6.4%)	1 (0.9%)	111 (99.1%)		0.16 (0.02–1.31)
Allied health—patient facing	222 (12.7%)	19 (8.6%)	203 (91.4%)		1.56 (0.67–3.62)
Facilities management/support services	25 (1.4%)	0	25 (100%)		0.00 (0.00–NaN**)
Medical doctor/doctor of osteopathy	523 (30.0%)	18 (3.4%)	505 (96.6%)		0.63 (0.27–1.47)
Nursing	716 (41.1%)	50 (7.0%)	666 (93.0%)		1.27 (0.59–2.75)

^†^Fisher’s Exact Test, otherwise, Chi Squared.

*Statistically significant when p-value < 0.05.

**NaN is Not a Number, unable to divide by 0.

Column percentages may not always add up to 100% due to missing data. Missing data ranged from 0.11–1.32%.

Administrative: Director, Supervisor, Manager, Chairs, Admission personnel.

Allied Health: Nonpatient facing: Lab personnel, Lab Technologist, Radiology.

Allied Health: Patient facing: Technologist, Medical Assistant, Speech Pathologist, Team Leader, Occupational Therapist, Dentist, Phlebotomy, Patient Education, Pharmacy, Other.

Facilities Management: Electrician, Environmental Services, Housekeeping, Support Staff.

Medical Doctor/ Doctor of Osteopathy: Attending, Resident.

Nursing: Registered Nurse, Advanced Practice Registered Nurse, Nurse Technicians.

### Seroprevalence

Table 1 shows selected demographics and clinical characteristics of the study participants by antibody status. Overall, 95 of 1743 were positive for SARS-CoV-2 IgG antibody corresponding to a hospital-wide HCW seroprevalence rate of 5.5%. Seropositive participants were more likely to be female and smokers, although not statistically significant. Individuals that identified as LatinX were significantly more likely to be seropositive [RR 1.79 (95% CI 1.02–3.14)]. Obese participants had a significantly increased risk of being seropositive [RR 1.97 (95% CI 1.25–3.32)]. The relative risk of being seropositive was higher among nurses [RR 1.27 (95% CI 0.59–2.75)] and patient facing allied health [RR 1.56 (95% CI 0.67–3.62)], but these findings did not reach statistical significance. Serious and chronic illness was not significantly associated with seropositivity (p = 0.89; data not shown).

The average day between RT-PCR and serology test date was 102 days for study participants, the range was 25–145 days. The distribution of SARS-CoV-2 IgG Days between RT-PCR and serology test date are show a detectable response at 145 days (Supplemental Figure 1). Among the 441 participants with a previous RT-PCR test, 85 (19.3%) were positive by RT-PCR. Of these, 64 (75.3%) were also seropositive by antibody testing but 21 (24.7%) were seronegative. 350 of 441 participants that had undergone diagnostic testing were negative by RT-PCR. Of these, 7 (2.0%) were seropositive. A total of 1302 participants had no prior RT-PCR test and of these participants, 23 (1.8%) were positive for SARS-CoV-2 IgG (Table 2).

**Table 2:** SARS-CoV-2 RT-PCR result by SARS-CoV-2 IgG status.

	Total n	SARS-CoV-2 IgG Status	
		Positive n (row %)	Negative n (row %)
Total	1743	95 (5.5%)	1648 (94.5%)
**RT-PCR tested**	441	72 (16.3%)	369 (83.7%)
Indeterminate/no result	6	1 (16.7%)	5 (83.3%)
Negative	350	7 (2.0%)	343 (98.0%)
Positive	85	64 (75.3%)	21 (24.7%)
**RT-PCR not tested**	1302	23 (1.8%)	1279 (98.2%)

We also report seropositivity status stratified by self-reported presence of COVID-19 symptoms. Seropositivity status was significantly associated with a self-report history of fever, sore throat, cough, shortness of breath, chills, myalgia, loss of appetite, loss of smell and taste, fatigue, and neurological signs (Table 3). Runny nose, nausea/vomiting, diarrhea, headache, rash, seizures, and altered consciousness were not associated with seropositivity of SARS-CoV-2 antibody status (Table 3). Seropositive participants, on average, had approximately 2 more symptoms relative to the seronegative participants.

**Table 3:** Self-Reported symptoms by SARS-CoV-2 IgG status.

Symptoms	Total n (column %)	SARS-CoV-2 IgG Status		p-value (all data)
		Positive n (row %)	Negative n (row %)	
Total	1743	95 (5.5%)	1648 (94.5%)	
Fever**	252 (14.5%)	43 (17.1%)	209 (82.9%)	p < 0.001^†^*
Sore throat***	608 (34.9%)	33 (5.4%)	575 (94.6%)	p < 0.001*
Cough	611 (35.1%)	45 (7.4%)	566 (92.6%)	0.02*
Runny nose	672 (38.6%)	32 (4.8%)	640 (95.2%)	0.39
Shortness of breath	280 (16.1%)	30 (10.7%)	250 (89.3%)	p < 0.001*
Chills	362 (20.8%)	43 (11.9%)	319 (88.1%)	p < 0.001^†^*
Nausea/vomiting	318 (18.2%)	16 (5.0%)	302 (95.0%)	0.83
Diarrhea	409 (23.5%)	26 (6.4%)	383 (93.6%)	0.43
Headache	869 (49.9%)	49 (5.6%)	820 (94.4%)	0.81
Rash	73 (4.2%)	3 (4.1%)	70 (95.9%)	0.79^†^
Muscle/joint pain	566 (32.5%)	56 (9.9%)	510 (90.1%)	p < 0.001*
Loss of appetite	285 (16.4%)	36 (12.6%)	249 (87.4%)	p < 0.001*
Loss of Smell/Taste	116 (6.7%)	44 (37.9%)	72 (62.1%)	p < 0.001*
Fatigue	815 (46.8%)	64 (7.9%)	751 (92.1%)	p < 0.001*
Seizures	5 (0.3%)	0	5 (100%)	1.00^†^
Altered consciousness	9 (0.5%)	2 (22.2%)	7 (77.8%)	0.08^†^
Other neurological signs	21 (1.2%)	5 (23.8%)	16 (76.2%)	0.0045^†^*

^†^Fisher’s Exact Test, otherwise, Chi Squared.

*Statistically significant when p-value < 0.05.

**Missing data included in analysis due to accounting for 5.2% of responses.

***Missing data included in analysis due to accounting for 5.3% of responses.

Column percentages may not always add up to 100% due to missing data. Missing data ranged from 0–1.03%.

Seropositive participants with a negative RT-PCR (n = 7), all reported symptoms (average of four symptoms), however, thee reported symptoms were variable from person to person. Whereas seronegative participants with a positive RT-PCR (n = 21) had an average of seven symptoms. Of the 23 seropositive study participants who did not undergo diagnostic testing, 12 (52.2%) reported fever, cough, fatigue, shortness of breath or loss of taste/smell. Seven (6.7%) seropositive study participants reported no COVID-19 symptoms and 5 (4.8%) reported less common symptoms of nausea/vomiting, diarrhea, headache, rash, or runny nose.

The majority of the participants (84.1–95.8%) reported following recommended hand hygiene practices and wearing the recommended personnel protective equipment (PPE) when being exposed to patients, body fluids, and/or patient surroundings. No statistical significance was observed in seropositivity status and hand hygiene practices or PPE usage. However, these same practices were not always followed among their peers. Lack of physical distancing among health care workers in work areas and break room was significantly associated with seropositivity (p = 0.05, p = 0.003, respectively) (Table 4).

**Table 4:** Infection prevention and control measures by SARS-CoV-2 IgG status.

	Total n (column %)	SARS-CoV-2 IgG Status		p-value (all data)
		Positive n (row %)	Negative n (row %)	
Total	1743	95 (5.5%)	1648 (94.5%)	
**Hand hygiene before and after patient contact?**				0.38^†^
Always	1598 (91.7%)	85 (5.3%)	1513 (94.7%)	
Most of the time	123 (7.1%)	9 (7.3%)	114 (92.7%)	
Occasionally	0	0	0	
Rarely	2 (0.1%)	0	2 (100%)	
**Hand hygiene after body fluid exposure?**				0.25^†^
Always	1670 (95.8%)	89 (5.3%)	1581 (94.7%)	
Most of the time	24 (1.4%)	3 (12.5%)	21 (87.5%)	
Occasionally	0	0	0	
Rarely	7 (0.4%)	0	7 (100%)	
**Hand hygiene after contact with patient surroundings?**				0.50^†^
Always	1466 (84.1%)	85 (5.8%)	1381 (94.2%)	
Most of the time	233 (13.4%)	8 (3.4%)	225 (96.6%)	
Occasionally	5 (0.3%)	0	5 (100%)	
Rarely	4 (0.2%)	0	4 (100%)	
**PPE when interacting with patients or their specimens?**				0.95^†^
Always	1549 (88.9%)	85 (5.5%)	1464 (94.5%)	
Most of the time	141 (8.1%)	8 (5.7%)	133 (94.3%)	
Occasionally	7 (0.4%)	0	7 (100%)	
Rarely	12 (0.7%)	0	12 (100%)	
**Physical distancing in work areas?**				0.05
Always	334 (19.2%)	13 (3.9%)	321 (96.1%)	
Most of the time	721 (41.4%)	33 (4.6%)	688 (95.4%)	
Occasionally	320 (18.4%)	21 (6.6%)	299 (93.4%)	
Rarely	351 (20.1%)	28 (8.0%)	323 (92.0%)	
**Physical distancing in break room?**				0.003*
Always	447 (25.6%)	14 (3.1%)	433 (96.9%)	
Most of the time	689 (39.5%)	37 (5.4%)	652 (94.6%)	
Occasionally	316 (18.1%)	15 (4.7%)	301 (95.3%)	
Rarely	222 (12.7%)	22 (9.9%)	200 (90.1%)	

^†^Fisher’s Exact Test, otherwise, Chi Squared.

*Statistically significant when p-value < 0.05.

Column percentages may not always add up to 100% due to missing data. Missing data ranged from 0.17–2.93%.

## Discussion

This is the first study, to our knowledge, that reports on the seroprevalence of HCWs in Boston, an area that was hard hit early on by the COVID-19 pandemic in the US. Serological testing allows us to assess the total proportion of asymptomatic and symptomatic individuals infected with SARS-CoV-2. Our results show a seroprevalence of 5.5% amongst HCWs at BMC. We identified 2% cases amongst HCW that were originally negative by RT-PCR testing. We also report a 1.8% prevalence amongst individuals that were not tested by RT-PCR. We report that seropositivity was associated with lack of physical distance in work areas and break rooms. Taken together, these results provide support for efficacy of hand hygiene, PPE and social distancing but also highlight the need to reinforce these practices when engaging with colleagues.

The present data suggest that racial disparities exist even among healthcare workers and support previous findings of higher odds of SARS-CoV-2 infection in LatinX individuals^18^. These disparities could be attributable due to inequities in social determinants of health such as, limited access to healthcare, socio-economic status, crowded housing conditions, and occupation^19,20^. Minority groups are disproportionately represented in essential work settings and have increased chances of being exposed to COVID-19^20^. There was suggestive evidence of a higher likelihood of being seropositive among patient-facing allied health care workers and nurses. This might be attributable to higher frequency and duration of exposure to COVID-19 patients. Alternatively, it could reflect increased hours working in environments where distancing is not possible or be reflective of community acquired transmission.

The seroprevalence of our institution (5.5%) was lower relative to Northwell Health Systems in New York Area and tertiary NYC hospitals (13.7 and 10%, respectively)^13,21^. Additionally, a recent meta-analysis estimated that prevalence of 7% (95% CI 4–11%) for SARS-CoV-2 antibodies amongst HCWs around the world^22^. A study among HCWs from 13 academic medical centers across the US reported a similar seroprevalence (6%) to ours^3^. The peak of cases in MA was slightly later than the peak in NY, and MA possibly had the advantage of being able to learn from the experience in NY. The governor of Massachusetts declared a state of emergency on March 10th, 2020, banned all public gatherings of more than 25 and closed all public schools on March 15th, 2020 and instituted a stay-at-home order on March 24th, 2020^23^. At the hospital level, BMC offered a diagnostic RT-PCR test on March 23rd, 2020, coupled with vigilant efforts to manage suspected COVID-19 exposure and wearing PPE^24^. In addition, universal masking policy for all people entering the hospital went into effect on March 27th, 2020. These early measures likely contributed to lower-than-expected COVID-19 infection rates in the hospital compared to other HCWs.

Whereas twelve seropositive HCWs never received a diagnostic test but reported sentinel symptoms of COVID-19, including fever, cough, fatigue, shortness of breath, loss of smell and taste. These individuals pose a risk of virus transmission as they are thought to have a period of viral shedding. Similar to previous findings, seropositive individuals experienced a wide-range of symptoms^22^. The prevalence of seropositive HCWs with no symptoms (n = 7) or mild symptoms (n = 5) (i.e. fever cough fatigue, shortness of breath, loss of smell/taste) was low but not zero at our institution. These individuals experienced mild symptoms and also pose a risk of virus transmission in their community and in the hospital. Studies suggest that asymptomatic individuals are a source of transmission, however, transmission dynamics remain to be elucidated^25–27^. One study reported that the viral load is found to be similar in symptomatic and asymptomatic patients while Zhou et al. demonstrated a lower viral load among asymptomatic cases^28,29^. Our results suggest that HCWs should be screened routinely with a symptom checklist and even one reported symptom should prompt diagnostic testing to help control SARS-CoV-2 spread in the workplace.

Nosocomial transmission cannot be ruled out even if there were no cluster outbreaks in the hospital. Encouragingly, most health care workers reported almost always following recommended hand hygiene measures and wearing PPE. The CDC found that detection of SARS-CoV-2 antibodies was less common among HCW who reported using PPE^1^. However, a remarkable increasing seropositivity trend was observed amongst HCWs that were always (3.9%), mostly (4.6%), occasionally (6.6%) and rarely (8.8%) able to physically distance in work areas (Table 4). A similar trend was also observed in seropositivity and HCWs who weren’t able to physically distance in break rooms. It remains unclear whether the observed prevalence was due to nosocomial or community acquired infection. However, the likelihood of community acquired infection is low as Boston regulations prohibited gatherings of more than 10 people from the start of the pandemic until July 2nd, 2020. In addition, mask coverings were required in public places at all times. This data highlights the need to promote infection prevention measures amongst co-workers to help mitigate infection rates and the need for further investigation to better understand transmission dynamics of SARS-CoV-2 amongst HCWs at BMC.

The dynamics of SARS-CoV-2 immunoglobulin durability is not completely known. It is unclear as to why RT-PCR positive with an average of seven symptoms were negative by SARS-CoV-2 IgG. The average time between RT-PCR and serology test for these individuals was 101 days and perhaps their levels started to wane. Most individuals became detectable for SARS-CoV-2 IgG levels by 14 days after infection but may wane as early as 3 months^30,31^. Our data supports that antibody response is quite variable from person to person and antibodies are detectable up to 145 days (Supplemental Figure 1). This variation could be attributable to various factors (i.e., age, disease severity, etc.). By conducting our sero-survey within two months of peak hospital and community prevalence, it negates the impact of delays in seroconversion and provides a better seroprevalence estimate. Additionally, the present study, benefited from a sensitive [93.8% (95% CI 82.80–98.69)] and specific [99.4% (95% CI 96.41–99.98%)] serological assay^32^.

Certain limitations are acknowledged. First, the study is limited by cross-sectional study design, which may underestimate or overestimate the seroprevalence. Second, this study utilized a qualitative antibody assay, whose sensitivity wanes with increased time post PCR, presumably secondary to waning antibody levels^33^. We intend to follow a subset of the participants to assess antibody durability over time. Some of our analyses were limited by small sample sizes which limit the interpretation of our findings. In addition, self-reported questionnaire data can lead to recall error, which could have led to underreporting or overreporting. Selection bias may also be present due to individuals with a belief and/or curiosity of concerning past infection. The study also suffered from under-enrollment of support staff at the hospital, even after targeted efforts for recruitment, which could affect the generalizability of results. Lastly, this study is limited to the hospital environment and not directly generalizable to the community setting.

In conclusion, we report a seroprevalence of 5.5% among HCWs at BMC. While these data cannot be utilized to make recommendations at an individual level, they can be used at a population level to reinforce the efficacy of personal hygiene, social distancing and PPE to prevent transmission. The importance of distance in the workplace at all times, even among our colleagues, is clear. Our findings suggest that aggressive efforts to protect our employees, including an early universal mask policy, were effective in protecting healthcare workers. Our data also highlight the presence of asymptomatic prevalence and support the need for studies characterizing transmissibility among asymptomatic individuals.

## Supplementary Information

Supplementary Figure 1.
