# Supplementary figures and images for: Seroprevalence of SARS-CoV-2 IgG antibodies and risk factors in health care workers at an academic medical center in Boston, Massachusetts
Source: Sci Rep. 2021 May 6;11:9694. doi: 10.1038/s41598-021-89107-5 (PMC8102593; doi:10.1038/s41598-021-89107-5)

**Supplemental Figure 1**. SARS-CoV-2 by Days between RT-PCR and Serology Test Date


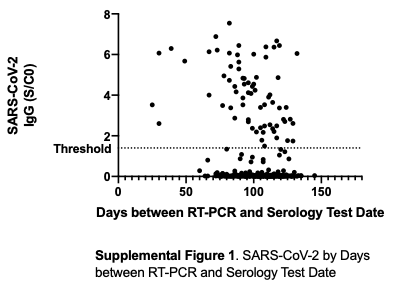

Supplement: Supplementary file 1 — Supplementary Figure 1. [file 41598_2021_89107_MOESM1_ESM.docx]
